# Evaluation of an in vitro model with a novel statistical approach to measure differences in bacterial survival of extended-spectrum *β*-lactamase-producing *Escherichia coli* on an inanimate surface

**DOI:** 10.1186/s13756-019-0558-7

**Published:** 2019-06-18

**Authors:** Veronica Weterings, Jacobien Veenemans, Amanda Kleefman, Marjolein Kluytmans-van den Bergh, Paul Mulder, Carlo Verhulst, Ina Willemsen, Jan Kluytmans

**Affiliations:** 1 Department of Infection Control, Amphia Hospital, P.O. box 90158, 4800 AK Breda, The Netherlands; 2 Medical Microbiology, Radboud University Medical Centre, P.O. box 9101, 6500 HB Nijmegen, The Netherlands; 3 Laboratory for Microbiology, Admiraal De Ruyter Hospital, P.O. box 15, 4460 AA Goes, The Netherlands; 4 Avans University of Applied Sciences, P.O. box 90116, 4800 RA Breda, The Netherlands; 5 Amphia Academy Infectious Disease Foundation, Amphia Hospital, P.O. box 90158, 4800 AK Breda, The Netherlands; 6 Julius Center for Health Sciences and Primary Care, University Medical Center Utrecht, Utrecht University, P.O. box 85500, 3508 GA Utrecht, the Netherlands; 7 Amphia Academy, Amphia Hospital, P.O. box 90158, 4800 AK Breda, The Netherlands; 8 Microvida Laboratory for Microbiology, Amphia Hospital, P.O. box 90158, 4800 AK Breda, The Netherlands

**Keywords:** Survival, *E. coli*, ST131, Environment, Persisters, ESBL

## Abstract

**Background:**

The role of environmental contamination in the transmission of Enterobacteriaceae is increasingly recognized. However, factors influencing the duration of survival in the environment have not yet been extensively studied. In this study, we developed and evaluated an in vitro model with a novel statistical approach to accurately measure differences in bacterial survival, that can be used to model the effects of multiple factors/conditions in future experiments.

**Methods:**

Two extended-spectrum β-lactamase (ESBL)-producing *Escherichia coli* (*E. coli*) isolates were used for this in vitro experiment: a CTX-M-15-producing *E. coli* sequence type (ST) 131 and a CTX-M-1-producing *E. coli* ST10 isolate. Each strain was 1:1 diluted in sterile water, sterile saline or sheep blood. Cover glasses (18 × 18 mm) were inoculated with the dilution and subsequently kept at room temperature. Bacterial survival on the glasses was determined hourly during the first day, once daily during the following 6 days, and from day 7 on, once weekly up to 100 days. The experiment was repeated six times for each strain, per suspension fluid.

**Results:**

Viable bacteria could be detected up to 70 days. A biphasic survival curve for all suspension fluids was observed, whereby there was a rapid decrease in the number of viable bacteria in the first 7 h, followed by a much slower decrease in the subsequent days.

**Conclusions:**

We found a difference in survival probability between *E. coli* ST10 and ST131, with a higher proportion of viable bacteria remaining after 7 h for ST131, particularly in sheep blood.

## Background

The important role of the environment in the nosocomial transmission of microorganisms is increasingly recognised [1–9]. As an example, several studies have demonstrated that prior room occupation by patients carrying *Acinetobacter baumannii*, vancomycin-resistent enterococci or methicillin-resistant *Staphylococcus aureus* increases the risk of nosocomial acquisition of these bacteria [3, 7–9].

A critical factor for transmission of a microorganism via the environment is its ability to survive on environmental surfaces. The ability of Gram-positive pathogens to survive on (hospital) inanimate surfaces for long periods of time has frequently been addressed [10–12]. Where the survival of Enterobacteriaceae on dry surfaces was long believed to be limited, recent studies have shown that environmental sources may contribute to the transmission of multidrug-resistant Enterobacteriaceae [13–15]. Havill et al. showed that carbapenem-resistant Enterobacteriaceae survived for extended periods of time on metal discs [16]. Also, other factors beside the ability to survive on dry surfaces have been reviewed [17], revealing that high humidity, low temperature, a higher inoculum and the presence of protein were associated with longer survival.

In infection control it is important to investigate what exactly are factors that support or hinder survival. The downside is, that it is difficult to accurately measure differences in bacterial survival, that can be used to model the effects of multiple factors or conditions (e.g. bio-based materials, antibacterial coatings) simultaneously.

We developed and evaluated a model to measure differences in bacterial survival of extended-spectrum β-lactamase (ESBL)-producing *Escherichia coli* on an inanimate surface, and assessed whether survival depended on sequence type and suspension fluid.

## Methods

Two ESBL-producing *E. coli* isolates from a well-documented Dutch collection of ESBL-producing Enterobacteriaceae were used for this in vitro experiment: a CTX-M-15-producing *E. coli* ST131 cultured from a patient during a large *E. coli* ST131 outbreak in a Dutch nursing home [18], and a CTX-M-1-producing *E. coli* ST10 isolate cultured from chicken meat [19]_*.*_

*E. coli* isolates were retrieved from − 80 °C Microbank vials (ProLab Diagnositcs, Ontario, Canada), and grown overnight at 35 to 37 °C on sheep blood agar (SBA) plates. For both *E. coli* isolates a one McFarland suspension was 1:1 diluted in sterile water, sterile saline and sterile sheep blood and the initial concentration of viable bacteria in these suspensions at time zero (t = 0) was determined for each material and strain separately following procedures described below. Cover glasses (18 × 18 mm) were inoculated with 20 μL of the bacterial suspensions. After inoculation, cover glasses were kept at room temperature. Bacterial survival on the cover glasses was determined hourly during the first 7 h, once daily during the first 7 days, and weekly from day seven until day 100. The cover glasses were placed into 2 mL brain-heart infusion (BHI) broth and vortexed for 30 s to suspend all viable bacteria present on the glasses. At each time point, bacterial survival was assessed for six cover glasses for each of the six bacterial suspensions.

For all bacterial suspensions (sheep blood, water and saline at t = 0 and BHI broth at the subsequent time intervals), a series of sheep blood agar (SBA) plates were inoculated with 20 μL of a 10-fold dilution series of the BHI-suspension. The number of colony-forming units (CFU) on the SBA plates was counted after overnight incubation at 35 to 37 °C. To enable accurate counting, the SBA plates with a colony count in the range of 30 to 300 CFU were used to estimate the number of viable bacteria in each suspension and on the cover glasses, and results were expressed as CFU per mL. When the colony count of all dilutions were below 30 CFU per plate, the remaining 1800 *μ*L of BHI-broth was filtered over a sterile 0.45 μm Millipore filter that was placed on a SBA plate. CFU on this plate were counted after overnight incubation at 35 to 37 °C. The detection limit of the assay was 56 CFU/mL.

### Statistical analysis

The dataset used for the statistical analysis consisted of a time-series of surviving concentrations of viable bacteria for each of 36 combinations formed by six repetitions, two strains and three suspension fluids. For each time-series the first concentration at t = 0 was deleted as the number of bacteria was too high to reliably measure (36 observations) and also concentrations equal to zero mainly in the upper tail of the time-series were deleted (233 observations). The number of remaining non-zero concentrations as of t = 1 (0.042 days) was 913 in total and ranged per time-series from 16 to 36 (average 25.4) across the 36 combinations. The day of the last non-zero concentration measured per time-series ranged from 18 to 70 (average 56.6) across the 36 combinations.

The number (C_t_) of viable bacteria surviving over time (t) was assumed to follow the exponential model C_t_ = C_0_*exp.(−rt) complying with a negative exponential distribution of survival time (days) with daily death rate(r). C_0_ denotes the concentration measured at 1 h (0.042 days). The model was reformulated after natural logarithmic transformation as ln(C_t_/C_0_) = −rt. The daily death rate r was assumed to change from r_1_ to a lower value r_2_ after 7 h (0.25 days) according to a broken-stick survival model with one change point set at 0.25 days. The effects of repetition, strain and suspension on r were estimated using linear mixed modelling through the origin. The effect of repetition (6 levels) was assumed to be random, whereas the effects of strain (2 levels) and suspension (3 levels) were assumed to be fixed and allowed to be different between r_1_ and r_2_. The within-repetition (co)variance matrix was assumed to have a first-order autoregressive structure.

The survival probability at 7 h was calculated as exp.(− 0.25*r_1_). During the first 7 h an hourly survival probability was calculated as exp.(−r_1_/24). After 7 h a daily survival probability was calculated as exp.(−r_2_).

Differences in r between strains and suspensions were estimated using the linear mixed model and exponentially transformed so as to be interpretable as ratios of hourly or daily survival probabilities. As both strain and suspension were in the model, their effects on survival were assessed independently. The statistical analyses were performed in SAS, version 9.2 (SAS Institute Inc., Cary, North Carolina, USA), and SPSS, version 23 (SPSS Inc., Chicago, Illinois).

## Results

**Fig. 1:**
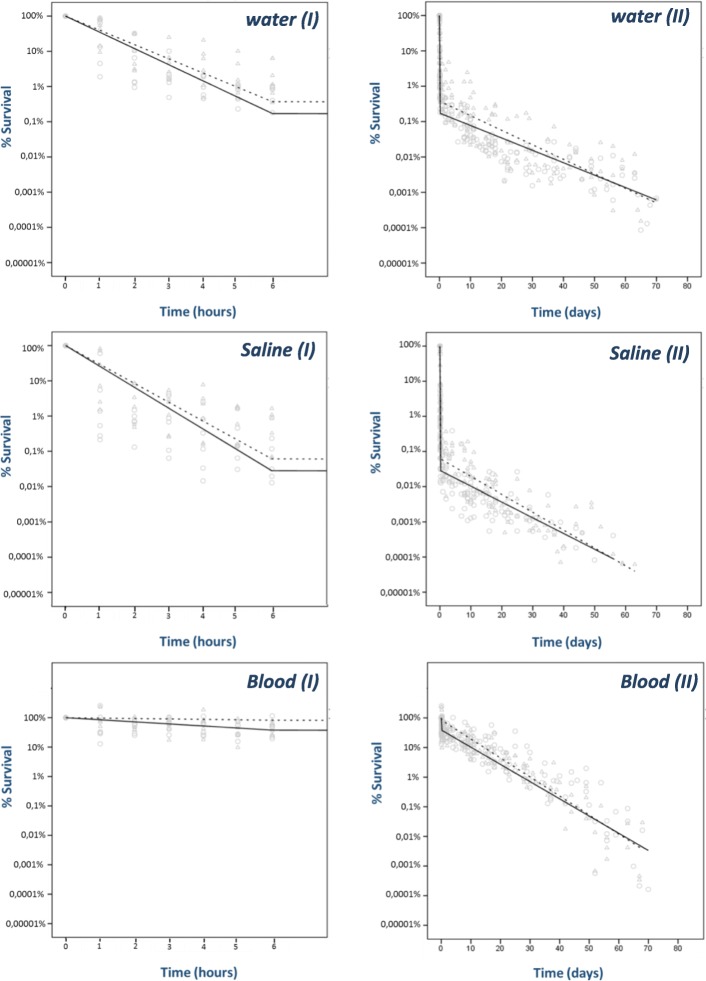
Observed (circle ST10; triangle ST131) and predicted survival of ST10 (solid line) or ST131(dotted line) in water, saline and sheep blood in the first 7 h (I) and total study period (II)

Survival curves of *E. coli* ST10 and ST131 in water, saline and sheep blood are shown in Fig. 1. A biphasic survival curve for all materials was observed, i.e. a rapid decrease in the number of viable bacteria was observed in the first 7 h, followed by a much slower decrease in the subsequent days.

In the first 7 h of the experiment, the hourly survival probability of *E. coli* ST131 was higher than that of *E. coli* ST10 (survival probability ratio 1.14 [95%CI: 1.05–1.25]) (Table 1). Also, the proportion of surviving bacteria in the first 7 h strongly depended on the suspension fluid used: the proportion surviving per hour was substantially higher in sheep blood than in the other media. (Table 1) In Table 2 the proportion of viable bacteria after 7 h is presented for all strain-suspension combinations. An adjusted survival probability ratio of 2.17 [95% CI: 1.37–3.44] was observed for *E. coli* ST131 as compared to *E. coli* ST10. The absolute difference in the proportion of viable bacteria after 7 h between ST types was largest for sheep blood (44.3%). After the first 7 h, the absolute number of viable bacteria remaining was very low (e.g. for ST131 in water, only 0,03% of the initial number of viable bacteria remained). At this time, the decline in the number of viable bacteria was less pronounced, and a difference between the strains no longer detectable **(**Table 1**).**

**Table 1:** Survival probabilities per hour (for time period t < 7 h) and per day (for time period t > 7 h) for *E. coli* ST10 and ST131 per suspension fluid. The survival probability ratio indicates the relative difference in survival between ST131 versus ST10

	t < 7 h			t > 7 h		
	Proportion surviving per hour (% [95% CI])		Survival probability ratio [95% CI]	Proportion surviving per day (% [95% CI])		Survival probability ratio [95% CI]
	*ST10*	*ST131*		*ST10*	*ST131*	
Water	25.6 [23.6–27.7]	29.1 [26.9–31.5]	1.14 [1.05–1.23]	90.2 [88.2–92.3]	89.0 [87.0–90.9]	0.99 [0.97–1.00]
Saline	34.6 [32.0–37.3]	39.3 [36.4–42.5]		92.2 [90.5–94.0]	91.0 [89.3–92.7]	
Blood	85.1 [78.8–91.9]	96.8 [89.6–104.5]		87.5 [85.8–89.1]	86.3 [84.6–87.9]

**Table 2:** Proportion surviving after 7 h for *E. coli* ST10 and ST131 per suspension fluid. The survival probability ratio indicates the relative difference in survival probability between ST131 versus ST10

	Proportion surviving after 7 h (% [95% CI])		Survival probability ratio [95% CI]
	*ST10*	*ST131*	
Water	0.03 [0.02–0.05]	0.06 [0.04–0.10]	2.17 [1.37–3.44]
Saline	0.17 [0.11–0.27]	0.37 [0.23–0.59]	
Blood	37.9 [23.9–60.1]	82.3 [51.9–100]	

## Discussion

The role of environmental contamination in the transmission of Enterobacteriaceae is increasingly recognized. However, factors influencing the duration of survival in the environment have not yet been extensively studied.

Our study showed that *E. coli* bacteria remained viable on dry inanimate surface up to 70 days and that survival of these bacteria, particularly in the first 7 h of the experiment, was influenced by the type of suspension fluid used. Survival was significantly prolonged in sheep blood as compared to water and saline. In addition, we showed a difference in survival probability between *E. coli* ST10 and ST131, particularly in sheep blood: at 7 h, the survival probability of ST131 was more than twice that of ST10 (82% versus 38%).

The enhanced survival in sheep blood is probably due to the presence of proteins and other nutrients creating an optimal environment for survival. This finding supports the notion that bacterial survival can be influenced by the degree of environmental contamination [20], and emphasises the importance of environmental cleaning to not only remove bacteria but also to get rid of nutrients for bacteria, as part of a effective infection control policy. Other studies previously reported extended survival times for *E. coli* [17, 21–23]. Neely showed that *E. coli* survived for extended periods of time (up to 15 days) on different hospital fabrics and plastic [22]. Starlander et al. reported extended survival (up to 28 days) and found a major difference in survival in the environment between different *E. coli* strains, whereby the ESBL-producing *E. coli* isolates tended to survive much longer than the AmpC-producing isolates. An explanation for this difference in survival was not described [21]. Also, in previous studies a range of materials was used to determine bacterial survival, e.g. Havill et al used metal disk to determine bacterial survival over time [16]. In our in vitro model we used an inert material to avoid any (chemical) influence of the material on the bacterial survival.

Our study has some limitations. First, a relatively high inoculum was applied on the cover glasses (~ 3 × 10^6^ CFU). Weber et al. and Neely both reported that bacterial survival can be affected by inoculum size [22, 23]. Neely suggested that bacteria in a nutrient-limiting situation can live on dying bacteria nearby, and therefore longer survival is expected in a more concentrated bacterial population. Secondly, the experiment was performed with only one isolate per ST type. Still, our results support the hypothesis that an enhanced environmental survival of ST131 may contribute to its potential to spread via (contaminated) environments more successfully than other clones. A recent study, however, describing an outbreak of ST131 in a Dutch nursing home did not find evidence of an increased acquisition risk of ST131 as compared to other ESBLs [24], and further work is required to investigate this hypothesis. Specifically, the in vitro experiment should be repeated and extended to include several different isolates, sequence types and varied inoculum sizes.

In our experiment, survival of ESBL-producing *E. coli* ST10 and ST131 followed a biphasic pattern, with a rapid decrease in the number of viable bacteria during the first 7 h, followed by a much slower decrease in the subsequent 70 days. The biphasic character of the survival curve may be explained by the fact that part of the suspended bacterial population consisted of so-called ‘persisters’. Bacterial persisters are rare, transient phenotypic variants in a non-growing state, that can tolerate environmental stresses (e.g. starvation, pH, antibiotics) and survive longer than the normal phenotypic variants. As mentioned above, this persister phenotype may explain the biphasic kill curve, whereby the first phase represents the rapid death of normal cells and the second phase indicates the remaining presence of persisters that are characterized by a slower cell death [25, 26]. Alternatively, the effect of environmental factors, and particularly the effect of drying, might be most pronounced during the first days of the experiment and contribute to the change in survival rate over time.

The ability of both strains to remain viable for prolonged periods of time on dry inanimate surfaces, underlines the importance that environmental cleaning is part of a comprehensive infection control policy.

In recent years, several novel products have been developed to reduce microbial contamination of the (hospital) environment, such as antimicrobial or “self-disinfecting” surfaces. Still, more research is necessary to determine their effectiveness in reducing microbial contamination. In this study, we developed a model with a novel statistical approach to accurately measure differences in bacterial survival, that can be used to model the effects of multiple factors/conditions simultaneously in future experiments.

## Conclusions

ESBL-producing *E. coli* ST10 and ST131 can survive on dry inanimate surfaces for long periods of time, where bacterial survival was increased in sheep blood as compared to water and saline. We showed a difference in survival probability between *E. coli* ST10 and ST131, with a higher proportion of viable bacteria remaining after 7 h for ST131, particularly in sheep blood.

## Data Availability

The datasets used and analysed during the current study are available from the corresponding author on reasonable request.

## Abbreviations

AmpC: Ampicillin-hydrolyzing cephalosporinase
CFU: Colony-forming units
CTX-M: Cefotaximase- Munich
*E. coli*: *Escherichia coli*
ESBL: Extended-spectrum β-lactamase
SBA: Sheep blood agar
ST: Sequence type
